# Cost-Effectiveness of a Specialist Geriatric Medical Intervention for Frail Older People Discharged from Acute Medical Units: Economic Evaluation in a Two-Centre Randomised Controlled Trial (AMIGOS)

**DOI:** 10.1371/journal.pone.0121340

**Published:** 2015-05-05

**Authors:** Lukasz Tanajewski, Matthew Franklin, Georgios Gkountouras, Vladislav Berdunov, Judi Edmans, Simon Conroy, Lucy E. Bradshaw, John R. F. Gladman, Rachel A. Elliott

**Affiliations:** 1 School of Pharmacy, University of Nottingham, Nottingham, United Kingdom; 2 Division of Rehabilitation and Ageing, University of Nottingham, Nottingham, United Kingdom; 3 Universty Hospitals of Leicester, Leicester, United Kingdom; Cardiff University, UNITED KINGDOM

## Abstract

**Background:**

Poor outcomes and high resource-use are observed for frail older people discharged from acute medical units. A specialist geriatric medical intervention, to facilitate Comprehensive Geriatric Assessment, was developed to reduce the incidence of adverse outcomes and associated high resource-use in this group in the post-discharge period.

**Objective:**

To examine the costs and cost-effectiveness of a specialist geriatric medical intervention for frail older people in the 90 days following discharge from an acute medical unit, compared with standard care.

**Methods:**

Economic evaluation was conducted alongside a two-centre randomised controlled trial (AMIGOS). 433 patients (aged 70 or over) at risk of future health problems, discharged from acute medical units within 72 hours of attending hospital, were recruited in two general hospitals in Nottingham and Leicester, UK. Participants were randomised to the intervention, comprising geriatrician assessment in acute units and further specialist management, or to control where patients received no additional intervention over and above standard care. Primary outcome was incremental cost per quality adjusted life year (QALY) gained.

**Results:**

We undertook cost-effectiveness analysis for 417 patients (intervention: 205). The difference in mean adjusted QALYs gained between groups at 3 months was -0.001 (95% confidence interval [CI]: -0.009, 0.007). Total adjusted secondary and social care costs, including direct costs of the intervention, at 3 months were £4412 (€5624, $6878) and £4110 (€5239, $6408) for the intervention and standard care groups, the incremental cost was £302 (95% CI: 193, 410) [€385, $471]. The intervention was dominated by standard care with probability of 62%, and with 0% probability of cost-effectiveness (at £20,000/QALY threshold).

**Conclusions:**

The specialist geriatric medical intervention for frail older people discharged from acute medical unit was not cost-effective. Further research on designing effective and cost-effective specialist service for frail older people discharged from acute medical units is needed.

**Trial Registration:**

ISRCTN registry ISRCTN21800480 http://www.isrctn.com/ISRCTN21800480

## Introduction

In the UK, early and rapid hospital triage of emergency patients is undertaken on acute medical units (AMU). Many patients in AMUs have a very short length of stay (< 1–2 days), [1] and are discharged home after assessment or a short period of stabilisation. More than 10% of all AMU attendees are frail older people, identified by the presence of one or more geriatric syndromes. [2, 3] Poor outcomes and high resource-use, observed for frail older people discharged from AMU, [2, 4] may be avoidable. [5, 6] There is strong evidence for the effectiveness of the process to manage frail older people known as Comprehensive Geriatric Assessment (CGA) in general [7, 8] but little evidence for this approach applied in the urgent care context. We therefore developed a specialist geriatric medical intervention for older people at risk of adverse outcomes following discharge from acute medical units, to facilitate CGA aiming to reduce the incidence of adverse outcomes and associated high resource-use. This intervention comprised geriatrician assessment of patients on the AMU and further short term community follow-up to continue the assessment and oversight of the delivery of medical and non-medical community interventions.

A randomised controlled trial (RCT) in two hospitals in Nottingham and Leicester assessed the effectiveness of this specialist geriatric medical intervention (AMIGOS trial). [6, 9] No effect of the intervention on patient outcomes at 90 days was shown: no significant differences between intervention and control group in days at home (primary outcome which took account of death, time spent in hospital, and any new care home placement), mortality, institutionalisation, dependency, mental wellbeing, and quality of life were found. [6]

The trial also showed no significant differences in health and social care resources use between groups. [6] However, the cost consequences and cost-effectiveness of the intervention in the AMIGOS trial have not previously been analysed. Our recent research provided innovative costing methodology to obtain accurate hospital cost estimates in the case of frail older people discharged from acute medical units, and to estimate both patient-level secondary and social care costs in the post-discharge period, [10] which we could apply to the economic evaluation alongside the AMIGOS trial.

There are several reasons for undertaking economic evaluation in the AMIGOS trial even though the intervention being tested showed no clinical benefit (no statistically significant advantages on its primary or secondary outcome measures). First, economic analyses in geriatric medicine, conducted from health and social care perspectives, are important in guiding allocation of resources in elderly care. For example, a recent RCT of a specialist hospital unit for people with delirium and dementia showed no statistically significant benefit on its primary outcome measure [11] and yet this unit was found to be cost-saving with a very high probability of cost effectiveness. [12] Second, subgroup economic analyses can identify potential subgroups of patients in whom an improved clinical intervention might be targeted and evaluated in an appropriately powered study. Third, the evidence of the economic impact of other CGA interventions is inconsistent [13, 14] [15]. Despite recommendations to assess opportunity costs, [16] only few include social care costs; the eight studies reporting costs in CGA trials in a recent review only reported costs from a hospital perspective, even though multiple health, social, private and voluntary agencies are involved in the care of frail older people which means that they did not consider whether costs were shifted to other areas of health care, or to social care or informal carers.[17]

The objective of this study was to assess cost consequences and cost-effectiveness of the specialist geriatric medical intervention compared to standard care, from the perspective of the UK National Health Service and publically funded personal social care. The trial-based economic evaluation is reported in accordance with the CHEERS Statement (S1 Appendix).

## Methods

### Clinical trial

A one to one parallel group individual patient RCT, Acute Medical Unit Comprehensive Geriatric Assessment Intervention Study (AMIGOS), was conducted. [6] The published trial protocol [9] and supporting CONSORT checklist are available as supporting information; see S1 Protocol and S1 Checklist, and the full report on the trial, including recruitment flow chart, is available elsewhere as open-access article.[6]

Patients were recruited in two hospitals in Nottingham (catchment population 675 000) and Leicestershire (catchment population 1.1 million), East Midlands, UK. Patients (aged 70 or over) at risk of future health problems (defined by a score of at least 2/6 on the Identification of Seniors At Risk tool [18–20]), discharged from acute medical unit (AMU) within 72 hours of attending hospital, were eligible. Patients were excluded if they were not resident in the hospital catchment area, as were those without capacity to give informed consent and where there was no consultee available. Baseline clinical data collection was by interview with a researcher collected from the patient, family members, or other informal or professional carers. Research staff, not involved in recruitment or baseline data collection, and blind to allocation, determined outcomes at 90 days (±7 days) after randomisation. Routine records were examined for mortality, changes of address, and readmission.

Standard care on the AMUs before recruitment for both the control and intervention groups was delivered, and comprised assessment and treatment by a consultant physician and attending medical team (and by a multidisciplinary team of physiotherapist, occupational therapist and nurse, if needed). Patients’ general practitioners were responsible for all aftercare. Participants in the control group received no additional intervention over and above standard care. The specialist geriatric medical intervention was delivered in the intervention group. Participants in the intervention group were assessed before discharge from the AMUs by a geriatrician, who delivered and coordinated the delivery of any additional immediate care or aftercare they deemed necessary (a review of diagnoses; a drug review; further assessment at home or in a clinic or by recommending admission rather than discharge; advance care planning; or liaison with primary care, intermediate care, and specialist community services). The intervention was expected to be complete within one month of randomisation. Further details of the intervention are described elsewhere.[6]

Between October 2010 and February 2012, 433 patients were recruited: 217 in the control group and 216 in the intervention group. Sixteen participants (11 in the intervention group) withdrawn at baseline after initial consent and were not included in clinical analysis at 90 days, [6] nor they were included in the cost-effectiveness analysis (cost data and status (dead or alive) at follow-up were not available for those patients). Therefore, in the trial-based economic evaluation, 212 standard care and 205 intervention group participants were analysed at 90-day follow-up (262/417 (63%) patients from Nottingham). Baseline characteristics of the population and clinical effectiveness outcomes have been reported previously.[6]

### Trial-based economic evaluation

#### Health effects

The health outcome for the cost-effectiveness analysis was quality adjusted life years (QALYs) gained, constructing utility values from the 3-level EuroQol-5D (EQ-5D-3L) [21] with societal weights. [22] Patient-reported EQ-5D-3L valuations at baseline and 90-day follow up (measuring health state on a scale in which 0 and 1 represent death and full health, respectively) were applied to estimate QALYs gained, assuming baseline utility until date of death for a patient dead at follow up. Therefore, a patient’s QALYs gained were calculated as the area under curve using linear interpolation between EQ-5D-3L measurement points, and health outcome was summarised into a single index. Other trial health status measures, [6] used in the imputation of missing self-reported EQ-5D-3L valuations, were: dependency in personal activities of daily living (Barthel ADL [23]), Charlson comorbidity score [24], Identification of Senior at Risk (ISAR) items and score [18–20], presence of geriatric conditions, and cognitive impairment (Mini-Mental State Examination (MMSE) [25]).

#### Costs

To estimate cost of delivering the intervention, the interaction time of the geriatrician was recorded for every patient that received the intervention, which included the duration of interaction for: initial assessment including all related activities; home visits (travel time was included); phone calls with the patient; and other patient related activities. Only the duration of time spent during clinic visits was not recorded, and so a time assumption was obtained from the PSSRU 2012. [26] The hourly cost of the geriatrician was estimated to be £132; this estimate includes cost aspects such as wages and on-costs, overheads, but not qualification costs, as described in the PPSRU 2012. [26] The intervention cost was calculated for each patient based on the recorded or assumed time spent by the geriatrician with the patient. Detail assumptions and calculations are presented in Table 1.

**Table 1:** Cost of specialist geriatric medical intervention.

**Type of interaction** ^a^	**Mean interaction duration (hours)** ^b^ **(median, min, max, 95% CI)**	**Mean cost of each interaction (£)** ^c^ ^d^ **(median, min, max, 95% CI)**
*Initial Assessment*	0.77 (0.75, 0.08, 5, 0.72–0.83)	99 (99, 0, 660, 91–106)
*Home visits*	0.56 (0, 0, 2, 0.46–0.65)	73 (0, 0, 264, 61–86)
*Phone calls*	0.25 (0.17, 0.03, 1, 0.21–0.3)	9 (0, 0, 132, 6–12)
*Clinic visits* ^e^	0.02 (0, 0, 0.57, 0.01–0.03)	3 (0, 0, 76, 1–5)
*Other activities*	0.18 (0, 0, 2.5, 0.14–0.23)	24 (0, 0, 330, 18–30)
***Mean intervention cost*** ^**f**^		***208 (186*, *0*, *660*, *192–227)***

^a^All of the interactions assume involvement of geriatricians’ time only.

^b^Time expressed in hours.

^c^Hourly wage based on the value of contract hour reported in PSSRU §15.5, p. 235 (PSSRU 2012 [26]), equal to £132.

^d^Equal to Mean duration of interaction × Hourly wage.

^e^It was assumed that clinic visits last 17.2 minutes as in PSSRU §10.8b, p. 183 (PSSRU 2012 [26]).

^f^In the full sample (205 patients in the intervention group). Mean costs in complete-case sample and in other subgroups analysed are presented in Table 3 and S3 Appendix.

Most health and social care services now use electronic administrative record systems to record patient care. Approvals were gained to obtain electronic administrative record systems data from hospitals and social care in both Nottingham and Leicester. In Nottingham, further approvals were obtained to gain access to general practices, ambulance services, and mental healthcare—the choice to access these extra services in Nottingham was due to the main research team for extracting data being located in Nottingham. The time requirements and logistics for obtaining access and extracting data also in Leicester were beyond the resource constraints of the study. Data were collected for three months post-hospital discharge (July 2009—March 2012). Based on our previous research,[10] extensive fieldwork was completed with the included agencies to derive parameters covering resource-use (details in S2 Appendix).

Hospital care data (day-case, inpatient, outpatient and intensive care) were obtained from two patient administration systems for patients that attended five hospitals in Nottingham. The Secondary Users Service (SUS) database was interrogated to obtain day-case, inpatient and outpatient data in Leicester—intensive care data was not available from this database.

Primary care resource-use data were obtained from Nottingham and Nottinghamshire GP practices. Of 84 GP practices serving our cohort, data were obtained from 53 practices (192/262 participants), coming from five electronic administrative record systems: SystmOne, 109 (57%); EMIS LV, 59 (31%); EMIS Web, 17 (9%); Synergy, 5 (3%); and EMIS PCS, 2 (1%), and were anonymised at the GP practice.

Ambulance service resource-use was extracted from the Caller Aided Despatch (CAD) IT service. The CAD system was cross-referenced with paper-based Patient Record Forms to identify participants (using participant name and place of pick-up). Data from mental healthcare services for older people were provided by the Nottinghamshire Healthcare Trust data via the RiO system. [27] Social care services within two different catchment areas in Nottingham (City and County), operating two different electronic systems, were identified. Services consisted of contacts and assessments, and care plans. Care plans included home, day, residential and telephone care, housing and meals-on-wheels. Similarly, social care data were collected from two catchment areas (City and County) in Leicester.

Unit costs for primary care services were applied based on time taken to perform each task using time assumptions obtained from PSSRU 2011/12, [28] empirical literature, or expert opinion, and mid-point yearly salary estimations taken from the NHS “Agenda for Change” pay rates. [29] Unit costs of hospital care were applied using national reference costs according to Healthcare Resource Group (HRG) case-mix. Inpatient spell costs were adjusted for length-of-stay using standard excess bed day costs. Unit costs for other services were obtained from PSSRU, standard Department of Health costs and other reference costs for the 2011/12 financial year. [26] The detail costing methods are described elsewhere, [10] and the sources of unit costs are presented in S2 Appendix.

Unit costs were combined with resource-use to generate patient-level costs. Patient-level secondary (inpatient, daycase, outpatient) and social care cost, collected for both sites, incurred during the trial period were calculated for all trial participants who remained in the study at 90-day follow-up (patients who died during the study were not classed as ‘withdrawn’). Critical care, ambulance service, Mental Health Trust, and primary care costs, omitted in the two-centre cost-effectiveness analysis, were analysed for the Nottingham sub-sample.

#### Statistical analysis

The economic evaluation adopted a secondary (inpatient, daycase, outpatient) and social care perspective. The incremental cost-effectiveness ratio (ICER) generated by the intervention over standard care was calculated using the following equation:

$$ICER=\frac{Cost_{Int}-Cost_{SC}}{QALY_{Int}-QALY_{SC}},$$

where *Cost*_*Int*_ (*Cost*_*SC*_) and *QALY*_*Int*_ (*QALY*_*SC*_) are mean cost and QALYs gained in the intervention (standard care) group, respectively. Patient cost and QALYs were adjusted by baseline characteristics using regression methods, pairwise bootstrapping with replacement was employed for adjusted patient costs and QALYs using 5000 replications, and the resultant incremental costs and QALYs were plotted on a cost-effectiveness plane. [30] Uncertainty around ICERs was investigated and cost effectiveness acceptability curves [31] [32] were constructed. The analyses were performed using STATA version 12 [33] and Microsoft Excel 2010.

Missing data for patient-reported EQ-5D-3L are: 84/417 (20.1%) baseline EQ-5D-3L, 106/417 (25.4%) follow-up EQ-5D-3L, resulting in QALYs obtained for 254/417 (60.9%) participants, including 16 (intervention: 8) dead at follow up. No statistically significant differences in the percentages of missing EQ-5D-3L and QALYs values between the intervention and standard care groups were observed: 43/205 (21.0%) vs. 41/212 (19.3%), *p* = 0.68, for baseline EQ-5D-3L; 45/205 (22.0%) vs 61/212(28.8%), *p* = 0.11, for follow-up EQ-5D-3L; and 78/205 (38.0%) vs 85/212 (40.1%), *p* = 0.67, for QALYs.

Missing EQ-5D-3L valuations were imputed using multiple imputation by chained equations, [34] incorporating the set of variables: age and sex; Charlson comorbidity (scores 2–3 and ≥4), 6 items of Identification of Senior at Risk (ISAR) tool, presence of geriatric conditions, cognitive impairment (Mini-Mental State Examination (MMSE) score), and permanent care home residence at baseline; Barthel Activities of Daily Living (ADL) score at baseline and follow-up, and hospital location; inpatient, day-case, outpatient, and social care costs. Forty five imputed datasets were generated; based on rule of thumb, number of imputations was equal to the percentage of patients with at least one variable in the imputation model missing, 45% (apart from missing EQ-5D-3L data, Barthel ADL scores were missing at baseline and follow up, 14% and 26%, respectively).

Secondary (inpatient, daycase, outpatient) and social care cost data, collected for both Nottingham and Leicester sites, and analysed in economic evaluation, were complete. In the Nottingham sample, cost data for all health services were complete, except primary care data missing for 70/262 (26.7%) patients.

In the full-sample cost-effectiveness analysis (CEA), imputed missing EQ-5D-3L valuations data were incorporated into generation of QALYs gained. Additionally, imputed baseline utility values were included as covariate in the adjustment models. Adjusted cost and QALYs were estimated controlling for age, sex, hospital location, and baseline utility (cost was additionally adjusted by Charlson co-morbidity (scores 2–3 and ≥4) and residence at care home). Adjustment models and diagnostic tests were calculated on each imputed dataset, to obtain adjusted cost and QALYs averaged across 45 imputations (Rubin’s rules), and to find the optimal generalized linear models (GLMs) for both costs and QALYs (considering the worst test result across imputations) [34]. Adjusted cost, calculated using the recycled prediction method, [30] and adjusted QALYs, obtained from ordinary least squares (OLS) regression, were used to generate cost-effectiveness plane and cost effectiveness acceptability curve on each imputed dataset. Full sample cost effectiveness acceptability curve was obtained from probability of cost-effectiveness for given willingness to pay, averaged across 45 imputations.

Complete-case CEA was undertaken, comprising 254/417 (60.9%) patients with complete QALY data. Adjusted cost and QALYs were estimated controlling for age, sex, and baseline utility, Charlson co-morbidity (scores 2–3 and ≥4), higher risk of future health problems (≥4 on Identification of Senior at Risk (ISAR) tool), and hospital location (cost was additionally adjusted by residence at care home at baseline). A diagnostic process was used to find the optimal GLM for both costs and QALYs; recycled prediction method [30] and OLS regression were used to generate adjusted patient cost and QALYs, respectively.

Cost analyses were conducted for the full sample (inpatient, daycase, and outpatient care) and for the Nottingham sample (incorporating complete-case resource-use dataset, 192/262 (73.3%) patients). Costs in the intervention and standard care arms, as well as incremental costs by services were estimated, handling uncertainty by non-parametric bootstrapping (95% confidence intervals around the point estimates were calculated using bias-corrected nonparametric bootstrap with 2500 replications).

The pre-planned subgroup analyses according to risk of future health problems (defined by ISAR score) and care home residence, as prognostically important baseline indicators of outcomes specified in the trial protocol (S1 Protocol), [9] were carried out. Cost analyses were conducted for moderate-risk patients defined as those with a score of 2≤ISAR<4 (all trial participants: 2≤ISAR≤6, higher-risk patients: ISAR≥4), and for care home residents: 254 (intervention: 122) and 108 (intervention: 52) participants, respectively. Net-benefit regression approach was applied to investigate subgroup effect on cost-effectiveness of the intervention, accounting for baseline characteristics and intervention-covariate interactions. [35] The patient-level net benefit (*nb*), calculated using the following equation,

nb(λ)=λE−C,

where *E* and *C* are the patient’s observed effect (QALYs) and total cost, respectively, was analysed in regression models for different willingness to pay thresholds (*λ*). Thus, the net monetary benefit (*NMB*) generated by the intervention over standard care,

$$\begin{array}{l} NMB(\lambda )=\underset{\text{mean}\;nb\;\text{in the intervention group}}{\underset{︸}{\left(\lambda QALY_{Int}-Cost_{Int}\right)}}-\underset{\text{mean}\;nb\;\text{in the standard care group}}{\underset{︸}{\left(\lambda QALY_{SC}-Cost_{SC}\right)}}\\ =\lambda \left(QALY_{Int}-QALY_{SC}\right)-\left(Cost_{Int}-Cost_{SC}\right), \end{array}$$

adjusted by age, sex, baseline utility, Charlson co-morbidity (scores 2–3 and ≥4), higher risk of future health problems (≥4 on ISAR tool), and no-care-home residence, with age- and utility-intervention interactions, was estimated for the whole group, and for the subgroups (incorporating additionally intervention-higher-risk (ISAR≥4) or intervention-no-care-home interaction terms in the regression, respectively). Cost effectiveness acceptability curves were drawn based on *p*-values for intervention coefficients in the regression models (reflecting the decision rule that the intervention should be implemented over standard care, at given threshold (*λ*), if *NMB* (*λ*) > 0). Net-benefit regression results for £20,000 threshold were presented.

Full sample net-benefit regression analysis was conducted, incorporating imputed missing EQ-5D-3L valuations data: diagnostic tests were calculated on each imputed dataset (considering the worst test result across imputations), Rubin’s rules were applied to obtain regression results over 45 imputation.[34]

## Results

### Intervention cost

In the full sample, mean per-patient cost of delivering the intervention was £208 (95% confidence interval [CI]: 192, 227). Initial assessments and home visits were major cost drivers of the intervention, with mean costs £99 (95% CI: 91, 106) and £73 (95% CI: 61, 86), respectively. Calculations are presented in Table 1.

### Full-sample using imputed data cost-effectiveness analysis

**Fig 1:**
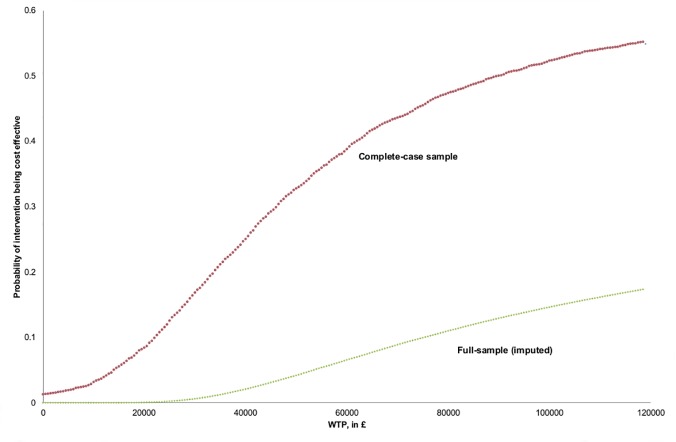
Cost-effectiveness acceptability curves (adjusted analyses, full sample). Full-sample CEAC is obtained from probability of cost-effectiveness for given WTP, averaged across 45 imputations.

In the full-sample cost-effectiveness analysis (CEA), 417 (intervention: 205) participants were analysed at 90-day follow-up, at which point 26 (intervention: 14) were dead. Comparing the intervention to control, mean total cost was non-significantly higher (£419, 95% CI: -597, 1371) and the difference in mean QALYs gained was 0.003 (95% CI: -0.012, 0.017). In adjusted cost-effectiveness analysis, total cost for the intervention group was significantly higher (£302, 95% CI: 193, 410) and QALYs gained difference was -0.001 (95% CI: -0.009, 0.007), with 0% probabilities of cost-effectiveness (ICER ≤ £20,000/QALY) and dominance. The probability that the intervention was dominated by standard care was 62%. (Table 2 and Fig 1).

**Table 2:** Full-sample cost-effectiveness analysis (mean cost in £ / mean QALY, 95% CI).

	Intervention (205 patients)	Standard care (212 patients)	Incremental cost / QALYs gained
The cost of care^a^	4267 (3697, 4934)	4057 (3367, 4882)	210 (-809, 1165)
The cost of care—adjusted^b^	4203 (4130, 4276)	4110 (4037, 4182)	94 (-10, 198)
The intervention cost	208 (192, 227)	0	208 (192, 227)
**Total cost (care cost + intervention cost)**	**4475 (3901, 5141)**	**4057 (3367, 4882)**	**419 (-597, 1371)**
***Total cost—adjusted (care cost adjusted + intervention cost)***	***4412 (4331*, *4490)***	***4110 (4037*, *4182)***	***302 (193*, *410)***
**QALYs gained**	**0.107 (0.097, 0.118)**	**0.103 (0.093, 0.112)**	**0.003 (-0.012, 0.017)**
***QALYs gained—adjusted*** ^**c**^	***0*.*106 (0*.*100*, *0*.*115)***	***0*.*107 (0*.*098*, *0*.*113)***	***-0*.*001 (-0*.*009*, *0*.*007)***
**ICER**			**£147 087/QALY**
***ICER adjusted***		***The intervention dominated by standard care***	

Multiple imputation by chained equation (MICE): predictive mean matching (*pmm*) for utilities and ordered logit (*ologit*) for Barthel ADL scores; 45 imputations.

^a^Inpatient, day-case and outpatient cost data were collected for both locations, Nottingham and Leicester.

^b^Adjusted by age, sex, hospital location (Leicester), and baseline utility, permanent care home residence, and Charlson co-morbidity (scores 2–3 and ≥4). A GLM model (family—gamma, link—0.8) was applied.

^c^ Adjusted by age, sex, hospital location (Leicester), and baseline utility. OLS was applied.

### Complete-case cost-effectiveness analysis

**Fig 2:**
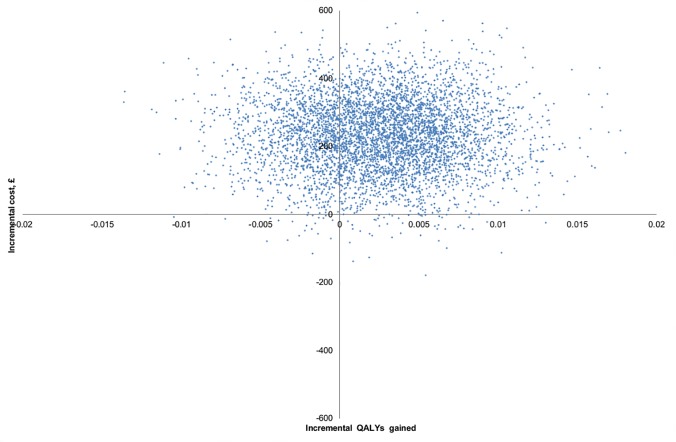
Cost-effectiveness plane—pairwise bootstrapping (adjusted analysis, complete-case sample).

In the subgroup of 254 (intervention: 127) patients with complete EQ-5D-3L data, including 16 (intervention: 8) patients dead at follow up, comparing the intervention and control groups, mean total cost was non-significantly higher (£228, 95% CI: -1203, 1527) and the difference in QALYs gained was 0.004 (95% CI: -0.012, 0.020). In adjusted cost-effectiveness analysis, total cost for the intervention group was significantly higher (£235, 95% CI: 21, 445) with QALYs gained difference equal to 0.002 (95% CI: -0.006, 0.011), ICER (£116,326/QALY, 95% CI: £13,900 to *∞*), and 1% probability of the intervention being dominant and 8% probability of ICER ≤ £20,000/QALY; the probability that the intervention was dominated by the control was 28%. (Table 3 and Fig 1 and 2).

**Table 3:** Complete-case cost-effectiveness analysis (mean cost in £ / mean QALY, 95% CI).

	Intervention (127 patients)	Standard care (127 patients)	Incremental cost / QALYs gained
The cost of care^a^	4114 (3417, 4956)	4085 (3145, 5297)	29 (-1386, 1333)
*The cost of care—adjusted* ^b^	*4139 (4004*, *4289)*	*4103 (3969*, *4252)*	*36 (-159*, *232)*
The intervention cost	199 (178, 217)	0	199 (178, 217)
**Total cost (care cost + intervention cost)**	**4312 (3612, 5153)**	**4085 (3145, 5297)**	**228 (-1203, 1527)**
***Total cost—adjusted (care cost adjusted + intervention cost)***	***4338 (4187*, *4490)***	***4103 (3957*, *4249)***	***235 (21*, *445)***
**QALYs gained**	**0.120 (0.108, 0.133)**	**0.117 (0.105, 0.129)**	**0.004 (-0.012, 0.020)**
***QALYs gained—adjusted*** ^**c**^	***0*.*125 (0*.*120*, *0*.*131)***	***0*.*123 (0*.*117*, *0*.*129)***	***0*.*002 (-0*.*006*, *0*.*011)***
**ICER**			**£63 559/QALY**
***ICER adjusted***			***£116 326/QALY*** ^**d**^

^a^Inpatient, day-case and outpatient cost data were collected for both locations, Nottingham and Leicester.

^b^Adjusted by age, sex, hospital location (Leicester), and baseline utility, permanent care home residence, Charlson co-morbidity (scores 2–3 and ≥4), and higher risk of future health problems at admission (≥4 on Identification of Senior at Risk (ISAR) tool). A GLM model (family—gamma, link—0.45) was applied.

^c^OLS was applied (adjustment covariates as above, except care home residence at baseline).

^d^From CEAC (Fig 1) we know that 95% CI for ICER is £13,900-*∞*.

### Cost analyses

In the full-sample of 417 (intervention: 205; Nottingham: 262), comparing the intervention and control groups, the cost of inpatient care was non-significantly lower (-£212, 95% CI: -1019, 537), day-case cost was significantly higher, £156 (95% CI: 36, 278), outpatient care costs was non-significantly higher, £46 (95% CI: -78, 167), and social care cost was non-significantly higher (£220, 95% CI: -270, 706), resulting in the cost of (secondary and social) care in the intervention group being non-significantly higher by £210 (95% CI: -809, 1156), and with an incremental total cost of £419 (95% CI: -597, 1371). (Table 4).

**Table 4:** Full-sample two-center cost analysis (mean cost in £, 95% CI).

	Intervention (205 patients)	Standard care (212 patients)	Incremental cost
Inpatient cost	1477 (1060, 2016)	1689 (1131, 2359)	-212 (-1019, 537)
Day-case cost	1134 (1047, 1233)	979 (908, 1061)	156 (36, 278)
Outpatient cost	470 (391, 563)	424 (345, 509)	46 (-78, 167)
***Total healthcare cost*** ^**a**^	***3081 (2636*, *3633)***	***3091 (2502*, *3806)***	***-10 (-860*, *763)***
Social care cost^b^	1186 (864, 1586)	966 (652, 1344)	220 (-270, 706)
***The cost of care***	***4267 (3697*, *4934)***	***4057 (3367*, *4882)***	***210 (-809*, *1165)***
The intervention cost	208 (192, 227)	0	208 (192, 227)
***Total cost (care cost + intervention cost)***	***4475 (3901*, *5141)***	***4057 (3367*, *4882)***	***419 (-597*, *1371)***

^a^Inpatient, day-case and outpatient cost data were collected for both locations, Nottingham and Leicester. The mean healthcare cost for the Nottingham sample was £3569 (95%CI: 3068, 4220), the mean healthcare cost for the Leicester sample was £2269 (95%CI: 1854, 2810), with healthcare cost significantly lower in the Leicester sample by -£1300 (95% CI: -2019, -516). This statistically significant difference may be attributed to significantly higher percentage of care home residents in the Leicester sub-sample (34.2% vs. 21.0%, p < 0.01), for whom healthcare cost was significantly lower than for non-residents in the whole sample (by -£880 (95%CI: -1631, -192)), to other non-observable differences between Leicester and Nottingham patient populations, as well as to different coding systems between sites (S2 Appendix). The two centre retrospective resource use datasets obtained for this study, and for related previous cost cohort study, [10] did not allow us to ascertain the latter hypothesis and explain fully the reasons of the difference in secondary care costs between Nottingham and Leicester.

^b^The mean social care cost for the Nottingham sample was £1010 (95%CI: 720, 1338), the mean social care cost for the Leicester sample was £1183 (95%CI: 770, 1652), with social care cost non-significantly higher in the Leicester sample by £173 (95% CI: -354, 726). Despite significantly higher percentage of care home residents in Leicester sample, for whom social care cost was higher than for non-residents in the whole sample (by £1026 (95%CI: 361, 1026)), social care cost in Leicester was not higher significantly and was not higher enough to reduce the overall difference in costs between sites. The reason could be that in the Leicester sample the percentage of patients living alone was significantly lower than in the Nottingham sample (31.0% vs. 46.6%, p < 0.01), and in the whole sample social care costs for those living alone was significantly higher by £804 (95%CI: 381, 1283), when comparing to those living with spouse. Social care costs was £366 (95%CI: 146, 585), £1170 (95%CI: 817, 1603), and £1835 (95%CI: 1209, 2517), for living with spouse, for living alone, and for care home residents, respectively.

In the subgroup of the Nottingham sample, comprising 192 (intervention: 95) patients with complete resource-use data, comparing the intervention to standard care, the cost of inpatient care was non-significantly lower (-£121, 95% CI: -1152, 1016), day-case care and outpatient costs were higher, £203 (95% CI: 55, 379) and £91 (95% CI: -969, 1324), respectively, and social care cost was significantly higher (£850, 95% CI: 120, 1606). The incremental cost for primary and tertiary care services, for which resource data were not collected for the Leicester sample, was -£12 (95% CI: -143, 119); primary care cost was non-significantly higher in the intervention group (£38, 95% CI: -25, 101). Comparing the intervention to standard care, the total care cost was non-significantly higher by £1010 (95% CI: -445, 2420), and the incremental cost was £1251 (95% CI: -211, 2650). (S3 Appendix, Table A).

### Subgroup and net monetary benefit analyses

Comparing the intervention to standard care in the subgroup of 254 (intervention: 122) participants who were classed as moderate-risk patients at baseline admission (<4 at ISAR tool), the inpatient cost was non-significantly lower (-£438, 95% CI: -1580, 668), day-case and outpatient care costs were higher, £146 (95% CI: 21, 282) and £53 (95% CI: -108, 223), respectively, and social care cost was non-significantly higher (£43, 95% CI: -430, 592), resulting in non-significantly lower cost of (secondary and social) care (-£196, 95% CI: -1487, 1060), and non-significantly lower total cost (-£6, 95% CI: -1295, 1251). (S3 Appendix, Table B). In the subgroup of 108 (intervention: 52) permanent care home residents at baseline, comparing the intervention to standard care, inpatient and social care costs were non-significantly lower (-£186 (95% CI: -1141, 765) and -£320 (95% CI: -1664, 948), respectively), resulting in non-significantly lower care cost (-£419, 95% CI: -2130, 1208), and non-significantly lower total cost (-£173, 95% CI: -1811, 1464). (S3 Appendix, Table C).

**Fig 3:**
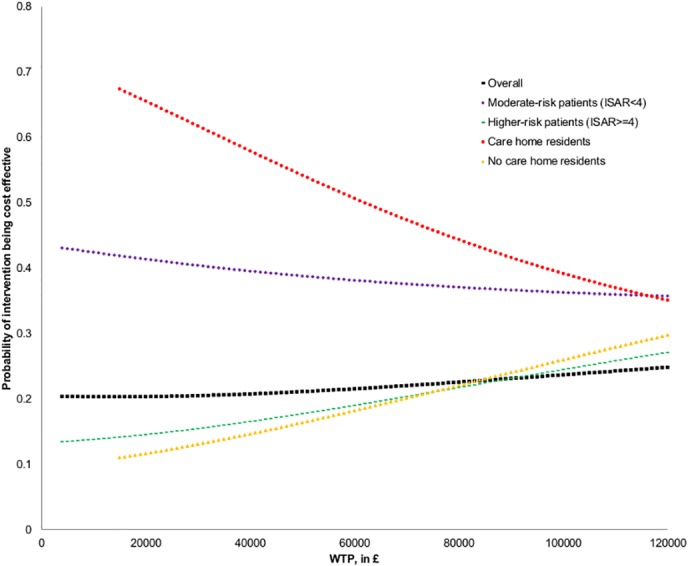
Cost-effectiveness acceptability curves (subgroups and overall, net-benefit regression approach). CEACs obtained from p-values, *p*, for intervention coefficient in net-benefit regressions for WTP≥£4,000 (for which Prob(>F)<0.05). In the case of negative coefficient, probability of cost-effectiveness is equal to *p*/2, in the case of positive, 1—*p*/2. Regression models analogical to those presented in Table 5, £1000-intervals for subsequent WTP applied. Due to model specification tests failed for WTP<£15,000, CEACs for care home subgroups are presented starting from WTP = £15,000.

In the whole group, at £20,000/QALY willingness to pay, comparing the intervention to standard care, the adjusted net monetary benefit (NMB) was -£423 (95% CI: -1425, 580; *p* = 0.41), with 20% probability of cost-effectiveness. Net-benefit regression showed significant association for utility at baseline (*p* = 0.01), and significantly higher net benefit for Leicester sub-sample compared to Nottingham sub-sample (£1278, 95% CI: 293, 2263; *p* = 0.01). The latter is the consequence of significantly lower healthcare costs in the Leicester sub-sample, compared with the Nottingham sub-sample (-£1300, 95% CI: -2019, -516) (see footnotes underneath Table 4 for further detail and explanation). Net benefit for higher-risk patients (ISAR≥4) was lower compared to moderate-risk patients (-£997, 95% CI: -62, 2057; *p* = 0.06) (Model 1 presented in Table 5 and Fig 3).

**Table 5:** Net-benefit regression models with treatment interactions.

Net-benefit regression model [at WTP = £20,000]	Model 1 Overall	Model 2 ISAR subgroups	Model 3 Care-home subgroups
	Coefficient (95% CI), in £; *p*-value^a^		
Age^b^	43 (-77, 163); 0.48	39 (-81, 160); 0.52	50 (-73, 173); 0.42
Baseline utility^b^	3089 (696, 5483); 0.01*	318 (794, 5583); 0.01*	2882 (405, 5360); 0.02*
Sex (female)	75 (-1046, 1196); 0.90	87 (-1032, 1205); 0.88	38 (-1092, 1169); 0.95
Charlson co-morbidity (scores 2–3)	-924 (-2229, 381); 0.16	-938 (-2244, 368); 0.16	-912 (-2217; 393); 0.17
Charlson co-morbidity (scores ≥4)	-1515 (-3574, 543); 0.15	-1514 (-3577, 550); 0.15	-1503 (-3556, 550); 0.15
Leicester location	1278 (293, 2263); 0.01*	1276 (291, 2261); 0.01*	1273 (285, 2260); 0.01*
High risk at ISAR tool (scores ≥4)	-997 (-62, 2057); 0.06	-656 (-2191, 880); 0.40	-988 (-2045, 69); 0.07
No care home residence at baseline	83 (-1230, 1397); 0.90	91 (-1226, 1408); 0.892	604 (-1205, 2413); 0.51
Intervention	-423 (-1425, 580); 0.41	-144 (-1446, 1158); 0.83^c^	437 (-1704, 2578); 0.69^d^
Intervention Age	-11 (-161, 138); 0.88	-6 (-155, 142); 0.93	-32 (-193, 129); 0.69
Intervention Baseline utility	1062 (-2295, 4419); 0.53	778 (-2672, 4227); 0.66	1568 (-2074, 5210); 0.40
Intervention High risk (ISAR≥4)		-707 (-2763, 1348); 0.50	
Intervention No care home residence			-1161 (-3696, 1375); 0.37
Constant	-1701 (-3386, -15); 0.04*	-1837 (-3589, -85); 0.04*	-2069 (-3934, -204); 0.03*

Multiple imputation by chained equation (MICE) estimates, 45 imputations (see Table 3). Interaction terms denoted by Intervention [.]. ISAR—Identification of Seniors at Risk.

*Significant at 0.05 level.

^a^Hubert-White *p*-values and 95% CI corrected for heteroskedasticity.

^b^Centred around mean.

^c^Intervention coefficient for identical regression model with ISAR dummy reversed (moderate-risk dummy (ISAR<4) instead of high-risk dummy (ISAR≥4)), -£852 (95% CI: -2435, 732; *p* = 0.29), indicates net monetary benefit (intervention vs. standard care) in the subgroup of high-risk patients.

^d^Intervention coefficient for identical regression model with care-home dummy reversed is -£724 (95% CI: -1915, 468; *p* = 0.23).

Net-benefit regression (at £20,000/QALY willingness to pay), according to risk of future health problems at admission (defined by ISAR tool) showed that, comparing the intervention to standard care: (i) in the subgroup of 254 (intervention: 122) moderate-risk patients (ISAR<4), the NMB was -£144 (95% CI: -1446, 1158; *p* = 0.83), with 41% probability of cost-effectiveness; (ii) in the subgroup of 163 (intervention: 83) higher-risk patients (ISAR≥4), the NMB was -£852 (95% CI: -2435, 732; *p* = 0.29), with 15% probability of cost-effectiveness. The NMB, generated by the specialist geriatric medical intervention over standard care, was non-significantly lower for high-risk patients, compared to moderate-risk patients (-£707, 95% CI: -2763, 1348; *p* = 0.50). (Model 2 presented in Table 5 and Fig 3).

Net-benefit regression (at £20,000/QALY willingness to pay), according to baseline care home residence showed that, comparing the intervention to standard care: (i) in the subgroup of 108 (intervention: 52) care home residents, the NMB was £437 (95% CI: -1704, 2578; *p* = 0.69), with 66% probability of cost-effectiveness; (ii) in the subgroup of 309 (intervention: 153) patients living in a non-institutional setting, the NMB was -£724 (95% CI: -1915, 468; *p* = 0.23), with 12% probability of cost-effectiveness. The NMB, generated by the intervention over standard care, was non-significantly lower for patients living in a non-institutional setting, compared to care home residents (-£1161, 95% CI: -3696, 1375; *p* = 0.37). (Model 3 presented in Table 5 and Fig 3).

## Discussion

### Summary of findings

This study confirmed that the specialist geriatric medical intervention for at risk older people discharged from acute medical units as tested in the AMIGOS study did not demonstrate benefits in health status, as no significant effect on QALY gain was observed. This study showed that the total cost for participants who received the intervention was higher than for those receiving standard care. The intervention was not cost-effective, and probability that it was dominated by standard care (more costs and less QALY) was high. Subgroup analyses did not identify any group in which there was a significant advantage of the intervention over the control, although there were trends towards greater cost-effectiveness of the intervention in care home residents and moderate-risk (but not high risk) patients (2≤ISAR<4). Comparing the intervention to standard care, cost savings for inpatient care and higher costs for social, day case and outpatient care were observed (however, these unadjusted cost differences were non-significant, except day case care).

### Strengths and limitations: internal validity

The strengths of this study were that it was conducted as an independent but pre-planned part of a RCT, [6, 9] and that resource-use data collection from various electronic systems and costing methods (tested previously in [10]) allowed accurate cost estimates to be produced. We applied EQ-5D-3L valuations to estimate QALYs due to its relevance for the UK policy makers. There were considerable EQ-5D-3L missing data at baseline and follow up, due to inability of frail older people, recently discharged from an acute medical care unit and in the post-discharge period, to complete EQ-5D-3L questionnaires. Additionally, 27% and 16% of participants had a prior dementia diagnosis and presented with cognitive impairment/confusion at baseline, respectively, [6] which is likely to have negatively affected the EQ-5D-3L questionnaire response rate. The large set of clinical measures collected as part of RCT enabled imputation methods to deal with missing EQ-5D-3L data. Economic evaluation of such complex services needs to consider broader outcomes than the QALY, however other health and well-being potential benefits of the intervention were investigated previously in the RCT, [6] and no benefits were shown. A weakness was that full resource-use data were collected from only one of the two centres of the AMIGOS study, thereby limiting the analysis to the secondary care and social service perspective and reducing the precision of the findings. However, it did not affect the main findings because primary and tertiary care accounted for 1% of the difference in total costs in the Nottingham sample. Another limitation was that the study sample was too small to conduct stratified cost-effectiveness analysis, and to provide convincing findings from subgroup analyses.

### Findings in context: external validity

AMIGOS was one of the few studies to examine the value of a specialist geriatric medical intervention to at risk patients on acute medical units (AMUs), and this is the only economic study of this approach. There is evidence that comprehensive geriatric assessment (CGA) is effective in older people, including acute care settings. [36, 37] However, the cost-effectiveness studies on CGA are limited, [13, 14] [15] and the economic value of CGA in the urgent care context was not yet known. The question how healthcare services can be configured to deliver high quality and efficient acute care for frail older people remains a challenge, and new forms of AMU services for older people which improve efficiency without adversely affecting mortality or re-admission rates merit further investigation. [38] The AMIGOS intervention was an attempt to deal with these needs, but despite no negative effect on mortality or re-admission, [6] the overall economic impact of this intervention was shown to be unfavourable.

### Interpretation

This study illustrates that although there are reasons to anticipate that specialist medical intervention might demonstrate clinical and economic advantages over standard medical care for at risk older people in acute medical units, these may not be seen when tested rigorously. The results presented here suggest there is a reasonable chance that standard treatment could be economically preferable. Thus, those developing, commissioning and providing such specialist services should carefully evaluate them and they should be seen as experimental until better models of care are developed that demonstrate robust evidence of effectiveness and cost-effectiveness. We had previously suggested that developments of this approach should consider targeting of the intervention upon higher-risk patients. [6] These results do not support this suggestion because there was a trend towards lower cost-effectiveness of the intervention in higher-risk group (ISAR≥4), compared to moderate-risk patients (2≤ISAR<4).

However, given the tendency towards better results in patients living in care homes, it is reasonable to consider developments of services focussed upon this group of patients and integrated with this sector of care. That is, in this sub-group of patients, the higher social care costs in the intervention arm were reversed to demonstrate some cost savings, resulting in favourable overall cost comparisons and a trend toward cost-effectiveness. This could be the consequence of the explicit intervention components of advance care planning or end of life care. These were more efficient for care home patients who were already primed for these events (more than patients living in their own homes). It is likely that, in the case of care home residents, social care resource use structure prior to the intervention made it possible to reduce spending as intended by other intervention components (social care maintenance costs), and keeping unchanged the costs that had already occurred (advance care planning). The (pre-planned) sub-group analysis (net benefit regression), supported by the above post hoc explanation of social care costs differences, suggests that the intervention targeted at patients admitted from care homes (or offered at care homes after a short stay at acute medical unit) might result in social care costs savings, overall cost reduction, and might be cost-effective. Hence, the specialist geriatric medical intervention is worth further evaluation in an appropriately powered study, in a larger population of care home residents.

Whilst the findings were not statistically significant, some savings were observed for inpatient care, and higher costs occurred for social, day-case and outpatient care, when comparing the intervention to standard care. The trade-off between inpatient stay and social care (combined with day-case hospitalizations and outpatient visits) was critical for the overall cost consequences and cost-effectiveness of the intervention. This observation demonstrates the need for inclusion of the social care perspective in economic evaluations of specialist services for older people, as reporting only hospital or secondary care costs would not depict the true economic impact of such services. The intervention was intended to limit the need for social care maintenance costs, but it did not reduce resource use in this sector, [6] as we showed higher overall costs occurred for social care in the intervention arm, resulting in unfavourable cost-effectiveness of the new service.

One of the explicit components of the intervention was to make use of outpatient and day case care to try to reduce subsequent health deterioration, the need for acute care, and readmissions. This study showed significantly higher costs of day case care and non-significantly higher costs of outpatient care, in the intervention arm, compared with standard care; the same patterns were observed for all sub-groups analysed as well. These findings are in line with the strategy of the delivery of medical interventions within the new model of care. Although some savings were observed for inpatient care (non-significant differences between groups), which might be the intended effect of more intensive use of day case and outpatient care, the overall economic impact of the intervention was negative and was driven by its effect on social care costs. The above discussion on the subgroup of care home residents, for whom the structure of social care spending is different, supports this notion.

This study provides evidence to guide resource use data collection for future economic evaluations of geriatric hospital services. The negligible impact of primary care on theoverall cost of the intervention suggests that resource use data from this sector of care might not be worth collecting and costing in future research; this process was found to be extremely time consuming and expensive and so this study provides the argument that more time and effort should be put into secondary and social care cost data. The large differences in secondary care costs between Nottingham and Leicester and different costs of the intervention between the two sites (suggested by the Nottingham sub-sample cost analysis for all services) confirm the value of the two-centre trial and justify the need for multicentre studies to obtain more generalisable results on cost-effectiveness.

## Supporting Information

S1 Checklist CONSORT checklist for AMIGOS clinical trial manuscript (Edmans J., Bradshaw E., Franklin M., Gladman J., Conroy S. “Specialist geriatric medical assessment for patients discharged from hospital acute assessment units: randomised controlled trial.” *BMJ* 2013;347:f5874 doi: 10.1136/bmj.f5874) (DOC)

S1 Protocol Judi Edmans, Simon Conroy, Rowan Harwood, Sarah Lewis, Rachel A Elliott, Philippa Logan et. al. “Acute medical unit comprehensive geriatric assessment intervention study (AMIGOS): study protocol for a randomised controlled trial.” *Trials* 2011, 12:200. (PDF)

S1 Appendix CHEERS Statement for the AMIGOS economic evaluation study (DOCX)

S2 Appendix Summary of resource-use parameters obtained in the AMIGOS study. (DOCX)

S3 Appendix Cost subgroup analyses. Table A. Cost analysis: resource-use data completed, Nottingham sub-sample (mean cost in £, 95% CI). Legend: Primary care, critical care, ambulance service and Mental Health Trust cost data were collected for Nottingham sample only. This cost analysis is conducted for Nottingham sample, for patients with resource-use data complete (cost data for all health services were complete, except primary care for which data were complete for 192/262 (73.3%) patients). Table B. Cost analysis: the subgroup of moderate-risk (ISAR < 4) patients (mean cost in £, 95% CI). Table C. Cost analysis: the subgroup of care home residents (mean cost in £, 95% CI)
(DOCX)

## References

[1] St Noble VJ, Davies G, Bell D. Improving continuity of care in an acute medical unit: initial outcomes. QJM. 2008 Jul;101(7):529–33. Epub 2008/04/18. eng. 10.1093/qjmed/hcn042 18417499

[2] Woodard J, Gladman J, Conroy S. Frail older people at the interface. Age Ageing. 2010;39(S1):i36.

[3] Ferguson C, Woodard J, Banerjee J, Conroy S. Operationalising frailty definitions in the emergency department-a mapping exercise. The Journal of Nutrition, Health and Ageing. 2009;13(Supplement 1):S266.

[4] Edmans J, Bradshaw L, Gladman JR, Franklin M, Berdunov V, Elliott R, et al The Identification of Seniors at Risk (ISAR) score to predict clinical outcomes and health service costs in older people discharged from UK acute medical units. Age Ageing. 2013 Nov;42(6):747–53. Pubmed Central PMCID: PMC3809718. Epub 2013/05/15. eng. 10.1093/ageing/aft054 23666405 PMC3809718

[5] Woodard J, Rowell G, Vartha R, Whittingham C, Vadher N, Conroy S. Appropriate prescribing in older people. J Nutr Health Aging. 2009;13(suppl 1):S477.

[6] Edmans J, Bradshaw L, Franklin M, Gladman J, Conroy S. Specialist geriatric medical assessment for patients discharged from hospital acute assessment units: randomised controlled trial. BMJ. 2013;347:f5874 Pubmed Central PMCID: PMC3793323. Epub 2013/10/10. eng. 10.1136/bmj.f5874 24103444 PMC3793323

[7] Beswick AD, Rees K, Dieppe P, Ayis S, Gooberman-Hill R, Horwood J, et al Complex interventions to improve physical function and maintain independent living in elderly people: a systematic review and meta-analysis. Lancet. 2008 Mar 1;371(9614):725–35. Pubmed Central PMCID: PMC2262920. Epub 2008/03/04. eng. 10.1016/S0140-6736(08)60342-6 18313501 PMC2262920

[8] Conroy S ST, Gladman J. The acute community—hospital interface: a mapping review. Medical Crises in Older People. Discussion paper series 2010.

[9] Edmans J, Conroy S, Harwood R, Lewis S, Elliott RA, Logan P, et al Acute medical unit comprehensive geriatric assessment intervention study (AMIGOS). Trials. 2011;12:200 Pubmed Central PMCID: PMC3184060. Epub 2011/08/26. eng. 10.1186/1745-6215-12-200 21864399 PMC3184060

[10] Franklin M, Berdunov V, Edmans J, Conroy S, Gladman J, Tanajewski L, et al Identifying patient-level health and social care costs for older adults discharged from acute medical units in England. Age and ageing. 2014 Sep;43(5):703–7. Epub 2014/07/26. eng. 10.1093/ageing/afu073 25059421

[11] Goldberg SE, Bradshaw LE, Kearney FC, Russell C, Whittamore KH, Foster PER, et al. Care in specialist medical and mental health unit compared with standard care for older people with cognitive impairment admitted to general hospital: randomised controlled trial (NIHR TEAM trial)2013 2013-07-02 10:40:13. 10.1136/bmj.f4132 PMC3698942 23819964

[12] Tanajewski LFM, Berdunov V, Gkountouras G, Harwood R, Goldberg S, Bradshaw L, Gladman J, Elliott RA. Cost effectiveness of a specialist Medical and Mental Health Unit for older people with cognitive impairment admitted to a general hospital: economic evaluation in a pragmatic randomised controlled trial (NIHR TEAM trial). 13th Annual International Conference on Health Economics, Management & Policy; Athens2014.

[13] Melis RJF, Adang E, Teerenstra S, van Eijken MIJ, Wimo A, Achterberg Tv, et al Multidimensional Geriatric Assessment: Back to the Future Cost-Effectiveness of a Multidisciplinary Intervention Model for Community-Dwelling Frail Older People. The Journals of Gerontology Series A: Biological Sciences and Medical Sciences. 2008 March 1, 2008;63(3):275–82. 10.1093/gerona/63.3.275 18375876

[14] Bouman A, van Rossum E, Evers S, Ambergen T, Kempen G, Knipschild P. Multidimensional Geriatric Assessment: Back to the Future Effects on Health Care Use and Associated Cost of a Home Visiting Program for Older People With Poor Health Status: A Randomized Clinical Trial in the Netherlands. The Journals of Gerontology Series A: Biological Sciences and Medical Sciences. 2008 March 1, 2008;63(3):291–7. 10.1093/gerona/63.3.291 18375878

[15] Philp I, Mills KA, Bhomra JT, Ghosh K, Long JF. Reducing hospital bed use by frail older people: results from a systematic review of the literature. International Journal of Integrated Care. 2013;13:e048 24363636 10.5334/ijic.1148 PMC3860583

[16] National Institute for Clinical Excellence. Guide to the methods of technology appraisal 2004 [updated 2006/05/15/].

[17] Ellis G, Whitehead MA, Robinson D, O’Neill D, Langhorne P. Comprehensive geriatric assessment for older adults admitted to hospital: meta-analysis of randomised controlled trials. BMJ. 2011 2011-10-27 17:08:35;343. 10.1136/bmj.d6553 PMC3203013 22034146

[18] McCusker J, Bellavance F, Cardin S, Trepanier S, Verdon J, Ardman O. Detection of older people at increased risk of adverse health outcomes after an emergency visit: the ISAR screening tool. J Am Geriatr Soc. 1999 Oct;47(10):1229–37. . Epub 1999/10/16. eng. 10522957 10.1111/j.1532-5415.1999.tb05204.x

[19] Dendukuri N, McCusker J, Belzile E. The identification of seniors at risk screening tool: further evidence of concurrent and predictive validity. J Am Geriatr Soc. 2004 Feb;52(2):290–6. . Epub 2004/01/20. eng. 14728643 10.1111/j.1532-5415.2004.52073.x

[20] McCusker J, Bellavance F, Cardin S, Belzile E, Verdon J. Prediction of hospital utilization among elderly patients during the 6 months after an emergency department visit. Ann Emerg Med. 2000 Nov;36(5):438–45. . Epub 2000/10/29. eng. 11054196 10.1067/mem.2000.110822

[21] EuroQol Group:EuroQol. A new facility for the measurement of health related quality of life. Health Policy. 1990;16:199–208. 10109801 10.1016/0168-8510(90)90421-9

[22] Dolan P. Modeling valuations for EuroQol health states. Med Care. 1997 Nov;35(11):1095–108. . Epub 1997/11/21. eng. 9366889 10.1097/00005650-199711000-00002

[23] Wade DT, Collin CL. The Barthel ADL index: a standard measure of physical disability? Int Dis Studies 1988;10:64–7. 10.3109/09638288809164105 3042746

[24] Cleves MA, Sanchez N, Draheim M. Evaluation of two competing methods for calculating Charlson's comorbidity index when analyzing short-term mortality using administrative data. J Clin Epidemiol. 1997 Aug;50(8):903–8. . Epub 1997/08/01. eng. 9291875 10.1016/s0895-4356(97)00091-7

[25] Folstein MF, Folstein SE, McHugh PR. ‘Mini-mental state’. A practical method for grading the cognitive state of patients for the clinician. Journal of Psychiatric Research 1975;12:189–98. 1202204 10.1016/0022-3956(75)90026-6

[26] Curtis L. Unit costs of health and social care 2012: Personal Social Services Research Unit; 2012.

[27] National Audit Office. The National Programme for IT in the NHS: an update on the delivery of detailed care records systems 2011 [10/11/12]. Available: http://www.nao.org.uk/publications/1012/npfit.aspx.

[28] Curtis L. Unit costs of health and social care 2012 University of Kent, Personal Social Services Research Unit, 2012.

[29] NHS Careers. Agenda for change—pay rates. 2011. Available: http://www.nhscareers.nhs.uk/working-in-the-nhs/pay-and-benefits/agenda-for-change-pay-rates/.

[30] Glick HA, Doshi JA, Sonnad SS, Polsky D. Economic Evaluation in Clinical Trials (Handbooks for Health Economic Evaluation). Oxford University Press, editor. Oxford 2007.

[31] Fenwick E, Byford S. A guide to cost-effectiveness acceptability curves. The British Journal of Psychiatry. 2005 August 1, 2005;187(2):106–8. 16055820 10.1192/bjp.187.2.106

[32] Fenwick E, Claxton K, Sculpher MJ. Representing uncertainty: the role of cost effectiveness acceptability curves. Health Economics. 2001;10:779–87. 11747057 10.1002/hec.635

[33] StataCorp LP. Stata data analysis and statistical Software. Special Edition Release 101 edition. 2008.

[34] White IR, Royston P, Wood AM. Multiple imputation using chained equations: Issues and guidance for practice. Stat Med. 2011 Feb 20;30(4):377–99. Epub 2011/01/13. eng. 10.1002/sim.4067 21225900

[35] Hoch JS, Briggs AH, Willan AR. Something old, something new, something borrowed, something blue: a framework for the marriage of health econometrics and cost-effectiveness analysis. Health Econ. 2002 Jul;11(5):415–30. . Epub 2002/07/12. eng. 12112491 10.1002/hec.678

[36] Baztan JJ, Suarez-Garcia FM, Lopez-Arrieta J, Rodriguez-Manas L, Rodriguez-Artalejo F. Effectiveness of acute geriatric units on functional decline, living at home, and case fatality among older patients admitted to hospital for acute medical disorders: meta-analysis. BMJ. 2009;338:b50 Pubmed Central PMCID: PMC2769066. Epub 2009/01/24. eng. 10.1136/bmj.b50 19164393 PMC2769066

[37] Fox MT, Persaud M, Maimets I, O'Brien K, Brooks D, Tregunno D, et al Effectiveness of acute geriatric unit care using acute care for elders components: a systematic review and meta-analysis. J Am Geriatr Soc. 2012 Dec;60(12):2237–45. Pubmed Central PMCID: PMC3557720. Epub 2012/11/28. eng. 10.1111/jgs.12028 23176020 PMC3557720

[38] Conroy S. Acute care for frail older people: time to get back to basics? Age Ageing. 2014 May 21. . Epub 2014/05/23. Eng. 24850539 10.1093/ageing/afu063

