# Supplementary material for: Cost-Effectiveness of a Specialist Geriatric Medical Intervention for Frail Older People Discharged from Acute Medical Units: Economic Evaluation in a Two-Centre Randomised Controlled Trial (AMIGOS)
Source: PLoS One. 2015 May 5;10(5):e0121340. doi: 10.1371/journal.pone.0121340 (PMC4420253; doi:10.1371/journal.pone.0121340)
Supplement: S2 Appendix — (DOCX) [file pone.0121340.s004.docx]

| **Appendix S2: Summary of resource-use parameters obtained in the AMIGOS study** | | | |
| --- | --- | --- | --- |
| **Service** | **Service parameter/code^a^** | **Resource-use source** | **Unit cost source^a^** |
| *Secondary care* |  |  |  |
| Inpatient and day case | Start of episode (date), end of episode (date), episode number, spell number, primary diagnosis (ICD-10 code and description), co-morbidities (ICD-10 code and description), procedures (OPCS-4 code and description), HRG-4 code, source of admission, method of admission, specialty on admission, method of discharge, destination of discharge, site code. | Patient Administration System (PAS)^b^; Secondary Users Service (SUS)^c^ | NHS reference costs 2011/12  (using HRG-4 codes) |
| Outpatient care | Start of episode (date), end of episode (date), type of visit, location, description, Treatment Function Code (TFC), TFC description, attendance status. | Patient Administration System (PAS)^b^; Secondary Users Service (SUS)^c^ | NHS reference costs 2011/12  (Using TFCs) |
| Intensive care | Start of episode (date), end of episode (date), level of care. | Patient Administration System (PAS)^b^ | NHS reference costs 2011/12 |
| *Primary care*^d^ |  |  |  |
| Event | Date of event, place of event, type of event, provider, free-text. | GP EAR’s system^b^ | PSSRU 2012  NHS wage schedule |
|  |  |  |  |
| Medication and wound dressings | Date of issue, rubric (name of drug), dosage, preparation, acute/repeat | GP EAR’s system^b^ | BNF 2012  C&D 2012 |
| *Ambulance service* |  |  |  |
|  | Date and time of event, call stop reason, call sign, resource type, hospital attended, clinic/ward attended by resource, primary complaint, dispatch code, government standard at time of call, time from call until arrival on scene, time at scene. | Caller Aided Dispatch (CAD) system, Patient Record Forms (PRF’s)^b^ | NHS reference costs 2011/12 |
| *Mental health care* |  |  |  |
|  | Activity date, activity type, activity code, specialty code, Team/Ward type. | CSE Healthcare RiO^b^ | NHS reference costs 2011/12 |
| *Social care* |  |  |  |
|  | Start date, end date, category of contact , service description, reason for referral, outcome of assessment, source | OLM Care First (City)^b^  Corelogic Frameworki (County)^b^  CareFirst (City)^c^  Social Services Information System (County)^c^ | PSSRU 2012 |

^a^If unit costs were sourced from a reference pre-2012 then these costs were standardised to 2012 prices using the Hospital & Community Health Services (HCHS) index [28, 39] for annual price inflation in the NHS.
^b^Resource-use source for Nottingham; ^c^ Resource-use source for Leicester

^d^Records extracted included consultations, procedures, telephone calls, home visits, administrative tasks, tests ordered and test results received. The protocol for identifying a participant’s GP practice, recruiting practices to the study, identifying participants, extracting data, the anonymisation process and attaching unit costs, and other details of primary care resource-use data collection, are available elsewhere [10] or upon request.
