# Supplementary material for: Cost-Effectiveness of a Specialist Geriatric Medical Intervention for Frail Older People Discharged from Acute Medical Units: Economic Evaluation in a Two-Centre Randomised Controlled Trial (AMIGOS)
Source: PLoS One. 2015 May 5;10(5):e0121340. doi: 10.1371/journal.pone.0121340 (PMC4420253; doi:10.1371/journal.pone.0121340)
Supplement: S3 Appendix — Legend: Primary care, critical care, ambulance service and Mental Health Trust cost data were collected for Nottingham sample only. This cost analysis is conducted for Nottingham sample, for patients with resource-use data complete (cost data for all health services were complete, except primary care for which data were complete for 192/262 (73.3%) patients). Table B. Cost analysis: the subgroup of moderate-risk (ISAR < 4) patients (mean cost in £, 95% CI). Table C. Cost analysis: the subgroup of care home residents (mean cost in £, 95% CI) (DOCX) [file pone.0121340.s005.docx]

**Appendix S3: cost subgroup analyses**

| **Table A. Cost analysis: resource-use data completed, Nottingham sub-sample (mean cost in £, 95% CI)** | | | |
| --- | --- | --- | --- |
|  | Intervention (95 patients) | Standard care  (97 patients) | Incremental cost |
| Inpatient cost | 1569 (932, 2515) | 1690 (1014, 2572) | -121 (-1152, 1016) |
| Day-case cost | 1175 (1053, 1342) | 972 (900, 1064) | 203 (55, 379) |
| Outpatient cost | 667 (532, 824) | 576 (455, 735) | 91 (-118, 275) |
| Secondary care cost | 3411 (2723, 4346) | 3238 (2490, 4208) | 172 (-969, 1324) |
| Primary care cost | 298 (249, 345) | 260 (221, 230) | 38 (-25, 101) |
| Critical care | 33 (0, 166) | 26 (0, 52) | 7 (-52, 114) |
| Ambulance service | 0 | 2 (0, 5) | -2 (-5, 0) |
| Mental Health Trust | 38 (14, 70) | 92 (37, 190) | -55 (-154, 9) |
| Primary and tertiary care cost^a^ | 369 (303, 469) | 381 (290, 490) | -12 (-143, 119) |
| ***Total healthcare cost*** | ***3779 (3096, 4733)*** | ***3617 (2864, 4551)*** | ***161 (-965, 1353)*** |
| Social care cost | 1375 (793, 2012) | 525 (177, 1017) | 850 (120, 1606) |
| ***The cost of care*^b^** | ***5155 (4198, 6268)*** | ***4145 (3221, 5173)*** | ***1010 (-445, 2420)*** |
| The intervention cost | 241 (219, 263) | 0 | 241 (219, 263) |
| ***Total cost***  ***(care cost + intervention cost)*** | ***5395*** ***(4446, 6526)*** | ***4145 (3221, 5173)*** | ***1251*** ***(-211, 2650)*** |

Primary care, critical care, ambulance service and Mental Health Trust cost data were collected for Nottingham sample only. This cost analysis is conducted for Nottingham sample, for patients with resource-use data complete (cost data for all health services were complete, except primary care for which data were complete for 192/262 (73.3%) patients).

^a^Primary care and critical care, ambulance and MHT (services for which data were collected for Nottingham sample only).

^b^Healthcare (inpatient, day-case, outpatient, EMAS, MHT, critical care, primary care) and social care cost.

**Table B. Cost analysis: the subgroup of moderate-risk (ISAR < 4) patients (mean cost in £, 95% CI)**

|  | Intervention  (122 patients) | Standard care (132 patients) | Incremental cost |
| --- | --- | --- | --- |
| Inpatient cost | 1348 (776, 2142) | 1786 (1110, 2799) | -438 (-1580, 668) |
| Day-case cost | 1076 (968, 1197) | 929 (864, 1003) | 146 (21, 282) |
| Outpatient cost | 518 (404, 648) | 465 (366, 588) | 53 (-108, 223) |
| ***Total healthcare cost*^a^** | ***2941 (2331, 3771)*** | ***3180 (2447, 4247)*** | ***-239 (-1449, 874)*** |
| Social care cost | 817 (487, 1244) | 773 (439, 1140) | 43 (-430, 592) |
| ***The cost of care*** | ***3758 (3065, 4700)*** | ***3953 (3122, 5117)*** | ***-196 (-1487, 1060)*** |
| The intervention cost | 190 (169, 210) | 0 | 190 (169, 210) |
| ***Total cost***  ***(care cost + intervention cost)*** | ***3948 (3257, 4888)*** | ***3953 (3122, 5117)*** | ***-6 (-1295, 1251)*** |
| ^a^Inpatient, day-case and outpatient cost data were collected for both locations, Nottingham and Leicester. | | | |

| **Table C. Cost analysis: the subgroup of care home residents (mean cost in £, 95% CI)** | | | |
| --- | --- | --- | --- |
|  | Intervention  (52 patients) | Standard care (56 patients) | Incremental cost |
| Inpatient cost | 1097 (581, 1816) | 1283 (665, 2115) | -186 ( -1141, 765) |
| Day-case cost | 1022 (892, 1172) | 976 (832, 1218) | 46 (-196, 251) |
| Outpatient cost | 264 (171, 380) | 223 (139, 345) | 41 (-110, 184) |
| ***Total healthcare cost*^a^** | ***2383 (1799, 3154)*** | ***2481 (1771, 3344)*** | ***-98 (-1110, 967)*** |
| Social care cost | 1669 (983, 2537) | 1989 (1098, 3193) | -320 (-1664, 948) |
| ***The cost of care*** | ***4052 (3076, 5164)*** | ***4471 (3320, 5880)*** | ***-419 (-2130, 1208)*** |
| The intervention cost | 245 (207, 285) | 0 | 245 (207, 285) |
| ***Total cost***  ***(care cost + intervention cost)*** | ***4297 (3236, 5359)*** | ***4471 (3320, 5880)*** | ***-173 (-1811, 1464)*** |
| ^a^Inpatient, day-case and outpatient cost data were collected for both locations, Nottingham and Leicester. | | | |
